# The Somatotropic Axis in the Sleep Apnea-Obesity Comorbid Duo

**DOI:** 10.3389/fendo.2020.00376

**Published:** 2020-06-12

**Authors:** Louis-Marie Galerneau, Anne-Laure Borel, Olivier Chabre, Marc Sapene, Bruno Stach, Janie Girey-Rannaud, Renaud Tamisier, Jean-Louis Pépin, Philippe Caron

**Affiliations:** ^1^Hypoxia PathoPhysiology (HP2) Laboratory, University Grenoble Alpes, Grenoble, France; ^2^Endocrinology Department, Pole Digidune, Grenoble Alpes University Hospital, Grenoble, France; ^3^Rivière Pneumology Center, Bordeaux, France; ^4^Tessier Clinic, Valenciennes, France; ^5^Pneumology Practice, Grenoble, France; ^6^Department of Endocrinology and Metabolic diseases, Pôle Cardiovascular and Metabolic, Larrey University Hospital, Toulouse, France

**Keywords:** obstructive sleep apnea, abdominal obesity, insulin-like growth factor, biomarker, cardiovascular risk

## Abstract

**Background:** Growth hormone (GH) stimulates the production of insulin-like growth factor 1 (IGF-1) in most tissues and together GH and IGF-1 profoundly impact adipose tissue deposition, glucose metabolism and cardiovascular function. A low serum IGF-I level has been reported as being associated with obstructive sleep apnea (OSA) and might be one of the mechanisms underlying cardio-metabolic risk in OSA patients.

**Methods:** In a multicenter national study, 817 patients consulting for suspicion of OSA (OSA confirmed for 567 patients) underwent serum IGF-1 measurements. We analyzed the association between an IGF-1 level below the median value of the population and variables related to cardio-metabolic risk: body mass index (BMI) and waist circumference, apnea hypopnea index (AHI), cholesterol and triglycerides (expressed as median and divided into quartiles for continuous variables).

**Results:** After adjustment for age and gender, low IGF-1 levels were associated with increased BMI and AHI (Odds ratios (OR) = 2.83; *p* < 0.0001 and OR = 3.03, *p* < 0.0001 for Quartile 4 vs. Quartile1, respectively), with elevated cholesterol levels (OR = 1.36, *p* = 0.0444), and elevated triglyceride levels (OR = 1.36; *p* = 0.0008).

**Conclusions:** Both adiposity and sleep apnea synergistically predict low levels of IGF-1 and thus could together contribute toward cardio-metabolic risk. Further work are needed to confirm whether IGF-1 levels allow grading severity and predicting response to treatments to aim at a personalized medicine for patients suffering from OSA.

## Introduction

Growth hormone (GH) stimulates the production of insulin-like growth factor 1 (IGF-1) in most tissues and together GH and IGF-1 profoundly impact adipose tissue deposition, glucose metabolism and cardiovascular function (1, 2). IGF-1 binds to the insulin receptor due to its high homology with insulin, but with a lower affinity, inducing “insulin-like” metabolic functions (3). GH deficiency induces low IGF-1 levels and is associated with cardio-vascular functional and morphological dysfunction, leading a higher mortality risk by cardiovascular disease (4–7). IGF-1 appears to be cardioprotective through maintenance of the functional and structural integrity of the microcirculation, increasing NO bioavailability, decreasing reactive oxygen species (ROS) production, and exerting anti-inflammatory, anti-apoptotic, and proangiogenic effects (3). It has also been demonstrated that low IGF-1 levels correlate with insulin resistance, atherogenic dyslipidemia, and increased blood pressure that constitute the three pillars of metabolic syndrome (8, 9). Moreover, low levels of IGF-1 have been found to be linked with the occurrence of late cardiovascular events and mortality (10, 11). Abnormalities of GH/IGF-1 axis with GH deficiency, are associated with cardio-vascular functional and morphological dysfunction and leads a higher mortality risk by cardiovascular disease.

Obstructive sleep apnea (OSA) is a highly prevalent condition that affects 4% of men and 2% of women in the general population, but is much more frequent in those with abdominal obesity (12). Sleep apnea is characterized by recurrent episodes of partial or complete obstruction of the upper airway during sleep, resulting in apneas and hypopneas with intermittent hypoxia, sleep fragmentation and a reduction in slow wave sleep (SWS) (13). The pituitary secretion of GH occurs preferentially at night (14) and IGF-1 levels positively correlate with the amount of SWS. Conversely, a decrease in sleep duration and an increase in indices of sleep apnea severity as measured by the apnea + hypopnea index (AHI) and the degree of oxygen desaturation, are associated with lower levels of GH/IGF-1. Obstructive sleep apnea alters insulin sensitivity and is independently associated with hypertension, coronary heart disease, stroke, arrhythmias, and all-cause mortality (13).

In a previous study by our team (13), IGF-1 was systematically measured in more than 800 men and women attending sleep clinics due to a suspicion of OSA. We reported that acromegaly, which consists in the abnormal excretion of GH by a pituitary adenoma, was more frequent in this population (15). In the present study, we took advantage of this well characterized prospective cohort to assess the GH/IGF-1 axis in the sleep apnea-obesity comorbid couple. We hypothesized that an OSA-related alteration of the somatotropic axis might be an independent mechanism underlying OSA-related insulin resistance and its cardio-metabolic consequences (16, 17).

## Materials and Methods

### Study Population

Between November 2013 and October 2014, patients referred for OSA to ten sleep centers in France were prospectively included. Inclusion criteria were age 18–75 years, and having a first assessment for OSA diagnosis. Exclusion criteria were any previously diagnosed sleep disorder, pregnant, or lactating women or patients under legal protection. After oral information about the study, each participant signed a written consent. The study was approved by the Grenoble Alpes University Hospital ethics committee (IRB: 13-CHUG-37) and registered in clinicaltrials.gov (NCT02789696). Eligible patients (1285) were asked to participate; 412 declined to do so or had exclusion criteria; 873 were included and 817 had complete laboratory data and were included in the analysis (15).

### Study Design

This was a prospective, cross-sectional, multicenter observational study.

### Data Collection

Each investigator completed the electronic record of the French sleep registry: the “*Observatoire du sommeil de la fédération de Pneumologie” (OSFP)*. This electronic health database, created by the French Federation of Pneumology, is a standardized electronic register used by sleep physicians in France to collect anthropometric characteristics, symptoms through validated scales for sleepiness, fatigue and depression, and medical history (smoking, hypertension, diabetes, hypercholesterolemia, hypertriglyceridemia, coronary heart disease, arrhythmias, stroke, heart failure, gastroesophageal reflux, depression, peripheral arteriopathy, glaucoma, and thyroid disease). Ethical committee approval for setting up the database was obtained from “Le Comité consultatif sur le traitement de l'information en matière de recherche en santé” (C.C.T.I.R.S n° 09.521) and authorization from the “Commission Nationale Informatique et Liberté” (C.N.I.L), the French information technology and personal data protection authority. The OSFP Independent Scientific Advisory Committee approved data use for this study. Beyond usual data items, a specific electronic file was created, including specific laboratory data (IGF-1, fasting lipid profile, fasting glucose) that were specifically measured for this study.

### Hormone Assays and Blood Tests

Blood samples were collected by the patient's closest medical analysis laboratory, at the patient's convenience. Serum IGF-1, fasting plasma glucose and fasting plasma lipid profile (total cholesterol, LDL cholesterol, HDL-cholesterol, and triglyceride levels) were measured with commercial kits available in each laboratory where a blood sample was collected.

### Nocturnal Poly(Somno)Graphy Data

Nocturnal polysomnography or polygraphy was performed using the routine clinical procedure of each participating sleep center. The recordings were scored according to standardized methods, described in American Academy of Sleep Medicine (AASM) Manual for Scoring Sleep and Associated Events (18). Patients with an AHI >15 apnea + hypopnea events per hour were considered as suffering from moderate to severe OSA.

### Statistical Analysis

Descriptive data are reported as median [interquartile range] for continuous variables and as n (%) for qualitative variables.

To assess which factors associated with low serum IGF-1 levels (defined as a level below the median value for the overall cohort), conditional univariate logistic regressions were performed since IGF-1 levels were not normally distributed. Univariate regressions were systematically adjusted on age and sex. Concerning continuous variables, if log-linearity was not respected, the variables were divided into quartiles, or recoded based on the median according to the *Akaike information criteria* (AIC) methodology. If log-linearity was respected the analysis was performed with continuous variables.

To look for the respective contributions of obesity and OSA to low IGF-1 levels, a multivariable logistic regression was performed with age, sex, body mass index (BMI), and AHI as independent variables and with IGF-1 level as a dependent variable. The same multivariable logistic regression was performed with waist circumference instead of BMI.

There was a maximum of 7% missing data for all data presented, except for 16% of missing data for the waist circumference. The missing data were not replaced for the univariate analysis. For the multivariate analysis, missing data were replaced by the median for the population for age, BMI and AHI, and the median according to sex for waist circumference.

## Results

The study population characteristics are shown in Table 1. The whole group was middle-aged, predominantly male, obese and suffering from the usual co-morbidities associated with sleep apnea.

**Table 1:** Characteristics of patients.

	***N* = 817**
**Anthropometric characteristics**	
Age, years	53 [44; 61]
Male gender, %	63.9
BMI, kg/m^2^	29.8 [26.1; 34.5]
Waist Circumference, cm	106 [97; 116]
**Sleep apnea**	
AHI, events/h	25.3 [15.0; 41.0]
AHI <5 events/h, %	7.2
AHI 5-15 events/h, %	17.7
AHI 15-30 events/h, %	32.8
AHI >30 events/h, %	42.2
Epworth Sleepiness score, /24	9 [5; 13]
**Laboratory measurements**	
IGF-1, ng/ml	138 [109; 176]
Fasting blood glucose, mg/dL	98 [91; 109]
Total cholesterol, g/l	2.04 [1.75; 2.30]
LDL cholesterol, g/l	1.24 [1.00; 1.46]
HDL cholesterol, g/l	0.51 [0.42; 0.61]
Triglycerides, g/l	1.21 [0.86; 1.74]
**Tabacco consumption**	
Former smokers, %	31.5
Current smokers, %	18.8
Hypertension, %	39.2
**Diabetes, %**	
All diabetes	7,6
type 1	2.9
type 2	4.7
**Dyslipidemia**	
Hypercholesterolemia, %	21.4
Hypertriglyceridemia, %	3.8
**Cardiovascular diseases**	
Coronary heart disease, %	2.6
Arrhythmias, %	4.8
Stroke, %	2.9
Heart failure, %	2.0
**Other comorbidities**	
Gastroesophageal reflux, %	18.3
Depression, %	11.0
Peripheral arteriopathy, %	1.7
Glaucoma, %	2.2
Thyroid disease, %	6.0

*Data are presented as median [interquartile range] for continuous variables and as percentage for qualitative variables. BMI, body mass index; AHI, apnea+hypopnea index; IGF-1 = Insulin-like growth factor-1*.

### Univariate Logistic Regressions

**Figure 1:**
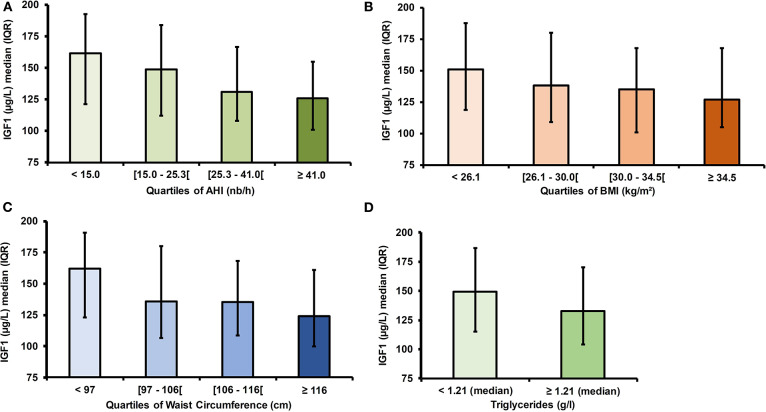
**IGF**-1 levels according to abdominal obesity and obstructive sleep apnea severity. **(A)** IGF-1 levels according to AHI quartiles. **(B)** IGF-1 levels according to BMI quartiles. **(C)** IGF-1 levels according to waist circumference quartiles. **(D)** IGF-1 levels according to triglyceride levels. IGF-1, Insulin Growth factor 1; AHI, Apnea Hypopnea Index; OR, Odds Ratio; IQR, interquartile range.

Higher age, BMI, waist circumference and AHI were found to be associated with IGF-1 levels below the median. Elevated triglycerides and total cholesterol levels were linked to low IGF-1 levels whereas fasting glucose levels, LDL-cholesterol and HDL-cholesterol were not (Table 2 and Figure 1).

**Table 2:** Factors associated with low IGF-1 levels (below the median) in univariate logistic regressions.

	**IGF-1 levels**	**IGF-1 levels**	**OR (95% CI)**	***p* value**
	**below the median**	**above the median**		
**Age (years)**	56 [48; 64]	49 [41; 59]		<0.0001
Q2 vs. Q1			1.70 (1.11; 2.61)	0.0143
Q3 vs. Q1			3.50 (2.30; 5.33)	<0.0001
Q4 vs. Q1			4.23 (2.76; 6.48)	<0.0001
**Male gender (%)**	62.5	65.2	0.90 (0.67; 1.21)	0.5003
**Anthropometric markers**				
**BMI (kg/m**^**2**^)*	30.8 [27.0; 34.9]	29.1 [25.1; 33.9]		<0.0001
Q2 vs. Q1			1.66 (1.08; 2.55)	0.0199
Q3 vs. Q1			1.83 (1.20; 2.80)	0.0051
Q4 vs. Q1			2.83 (1.82; 4.41)	<0.0001
Waist circumference (cm)*	109.0 [100.0; 119.0]	104.0 [93.0; 114.0]		<0.0001
Q2 vs. Q1			2.09 (1.29; 3.38)	0.0026
Q3 vs. Q1			2.25 (1.40; 3.63)	0.0009
Q4 vs. Q1			3.01 (1.85; 4.89)	<0.0001
**Sleep apnea severity**				
AHI (events/hour)*	30.0 [18.0; 49.0]	22.0 [10.7; 35.0]		<0.0001
Q2 vs. Q1			1.02 (0.65; 1.61)	0.9304
Q3 vs. Q1			1.80 (1.14; 2.84)	0.0116
Q4 vs. Q1			3.03 (1.88; 4.88)	<0.0001
Epworth score*	9 [5; 12]	9 [6; 13]		0.7535
Q2 vs. Q1			1.20 (0.77; 1.87)	0.4168
Q3 vs. Q1			1.28 (0.82; 2.00)	0.2843
Q4 vs. Q1			1.18 (0.75; 1.86)	0.4658
**Plasma glucose and lipid profile**				
Glucose (mg/dL)	101 [92; 114]	96 [90; 105]	1.33 (0.97; 1.82)	0.0775
Total cholesterol (g/L)	2.08 [1.78; 2.34]	2.01 [1.73; 2.26]	1.36 (1.01; 1.84)	0.0444
HDL-c (g/L)	0.51 [0.42; 0.61]	0.50 [0.42; 0.61]	0.68 (0.26; 1.77)	0.4286
LDL-c (g/L)	1.25 [0.98; 1.48]	1.22 [1.01; 1.44]	1.30 (0.96; 1.75)	0.0928
Triglycerides (g/L)	1.35 [0.95; 1.91]	1.14 [0.79; 1.60]	1.36 (1.13; 1.62)	0.0008

*Data are reported as median [interquartile range] for continuous variables and by percentage for qualitative variables in the subgroups of participants having IGF-1 below or above the median. Univariate logistic regressions with IGF-1 levels as dependent variable are reported by Odd ratios (95% CI) and are adjusted for age and gender. BMI, body mass index; Q, quartile; AHI, apnea+hypopnea index*.

### Multivariate Analysis

A multivariable regression analysis was performed with IGF-1 levels as dependent variable and age, sex, BMI and AHI as independent variables. Age, BMI and AHI were independently associated with IGF-1 levels (Table 3).

**Table 3:** Respective contribution of age, gender, BMI, and apnea severity to low IGF-1 levels in multivariate analysis (below the median).

	**OR (95%CI)**	***p* value**
**Age**		**<0.0001**
Q2 vs. Q1	1.65 (1.06; 2.58)	0.0269
Q3 vs. Q1	3.46 (2.23; 5.37)	<0.0001
Q4 vs. Q1	4.04 (2.59; 6.33)	<0.0001
**Male gender**	0.76 (0.55; 1.06)	**0.1065**
**BMI**		**0.0022**
Q2 vs. Q1	1.58 (1.03; 2.42)	0.0383
Q3 vs. Q1	1.75 (1.14; 2.69)	0.01
Q4 vs. Q1	2.36 (1.51; 3.69)	0.0002
**AHI**		**0.0003**
Q2 vs. Q1	0.99 (0.63; 1.56)	0.9609
Q3 vs. Q1	1.59 (1.04; 2.43)	0.0331
Q4 vs. Q1	2.39 (1.51; 3.79)	0.0002

*A multivariable logistic regression model was built with IGF-1 levels as dependent variable and age, gender, BMI, AHI as independent variables. Results are reported by odds ratios (95% CI). BMI, body mass index; AHI, apnea+hypopnea index; Q, quartile. The bold values indicate p value corresponding to the variable as a whole*.

Sleep apnea syndrome is associated with a central distribution of adiposity that was not captured by BMI. To take into account this potential confounding factor in the association between AHI and IGF-1 levels, we repeated the multivariable model including waist circumference instead of BMI among the independent variables. Results were similar to the previous model (Table 4).

**Table 4:** Respective contribution of age, gender, waist circumference, and apnea severity to low IGF-1 levels (below the median).

	**OR (95%CI)**	***p* value**
**Age**		** <0.0001**
Q2 vs. Q1	1.6 (1.03; 2.49)	0.0375
Q3 vs. Q1	3.23 (2.09; 4.98)	<0.0001
Q4 vs. Q1	3.61 (2.33; 5.58)	<0.0001
**Male gender**	0.71 (0.51; 0.99)	**0.0419**
**Waist circumference**		**0.0006**
Q2 vs. Q1	1.98 (1.28, 3.06)	0.0023
Q3 vs. Q1	1.5 (0.97; 2.33)	0.0719
Q4 vs. Q1	2.42 (1.55; 3.77)	0.0001
**AHI**		** <0.0001**
Q2 vs. Q1	1.04 (0.66; 1.64)	0.8581
Q3 vs. Q1	1.65 (1.08; 2.53)	0.0199
Q4 vs. Q1	2.57 (1.62; 4.06)	<0.0001

*A multivariable logistic regression model was built with IGF-1 levels as dependent variable and age, gender, waist circumference, AHI as independent variables. Results are reported by odds ratios (95% CI). AHI, apnea+hypopnea index. The bold values indicate p value corresponding to the variable as a whole*.

## Discussion

In this large prospective cohort, low IGF-1 levels were independently associated with increasing age, waist circumference or BMI and AHI. The originality of our data is to demonstrate that severe sleep apnea was the single comorbidity associated with low IGF-1 values, independent of BMI or waist circumference. Thus, both adiposity and sleep apnea seem to synergistically predict low levels of IGF-1 and thus could participate together toward cardio-metabolic risk.

### IGF-1 Levels in Obesity, Metabolic Syndrome and Cardiovascular Diseases

Plasma IGF-1 levels are known to be inversely correlated with BMI particularly in individuals with central obesity (1). The novelty of our work is to demonstrate that sleep apnea is a key player in the GH/IGF-1 axis dysfunction occurring in obesity. Low IGF-1 levels were also found to be associated with the different components of metabolic syndrome, type 2 diabetes, and cardiovascular diseases (1, 9, 19). Results from the Framingham heart study showed a dose-response relationship between lowering in IGF-1 values and the number of abnormalities of metabolic syndrome factors (8, 20). Accordingly, normal IGF-1 levels were linked with a lower prevalence of metabolic syndrome (21). The results of the present study are in agreement with these previous results since in univariate analysis increased levels of triglycerides were associated with low IGF-1 levels.

Low IGF-1 levels have previously been linked with reduced insulin sensitivity, and with a higher risk of glucose intolerance and type 2 diabetes (22–24). Low IGF-1 levels were linked to coronary atherosclerosis and re-stenosis (25–27), ischemic heart disease (11, 28, 29), congestive heart failure (30), and stroke (31–33). In the current study, we found no significant association between low IGF-1 and fasting glucose, type 2 diabetes, hypertension or previous cardiovascular events. However, the prevalence of previous cardiovascular diseases was relatively low among included patients and the absence of a significant association might be explained by lack of power.

### IGF-1 and OSA

As well as chronic intermittent hypoxia, OSA also induces sleep fragmentation and an inability to achieve deep sleep that alter circadian hormone regulation, impacting the corticotropic and somatotropic axis. Somatotropic cells of the anterior pituitary secrete GH mostly during SWS (14, 34, 35). Thus, longer SWS has been associated with increased IGF-I levels (36) whereas poorer sleep quality or OSA, by reducing SWS, inhibits the somatotropic axis (37). IGF-1 levels vary with age and the picture of the relationship is further complicated by the fact that both OSA and age are impacting the amount of SWS. The specific role of somatotropic axis dysfunction in OSA deserves attention since part of the OSA related cardiometabolic risk might be explained by lower IGF-1 levels in apneic patients. An association between OSA and lower GH and IGF-1 levels has been previously reported (38–42), independent of obesity (43, 44). However, the sample size of previous studies generally did not allow adjustment for confounders and multivariate analysis. In contrast, our large sample made it possible to demonstrate an inverse dose-response relationship between IGF-1 levels and the severity of AHI, independently of age, BMI or waist circumference. Circulating IGF-I and its interaction with IGFBP-1 is crucial for glucose homoeostasis and exert a protective effect against the development of glucose intolerance (22). On the other hand, there is a bidirectional association between both type 1 and 2 diabetes and obstructive sleep apnea (45–48). Mechanistic studies in rodent exposed to intermittent hypoxia have demonstrated organ specific insulin resistance related to intermittent hypoxia (49–51). Namely, we have evidenced that IH induces a pro-inflammatory phenotype of the adipose tissue, which may be a crucial link between OSA, central obesity and the development of insulin resistance (51). The association in human literature between IGF-1 and insulin resistance is more conflicting but seem to exist in non-diabetic patients with sleep apnea (52). The interaction between IGF-1, central obesity and insulin resistance is difficult to dissect precisely in clinical studies but is certainly playing a major role in OSA-related cardio metabolic co-morbidities (43, 53). Thus, low IGF-1 levels could be considered as a biomarker of OSA severity toward cardiometabolic health, being specifically related to OSA and associated with an increased cardiometabolic risk.

The standard therapy for OSA is continuous positive airway pressure (CPAP), but responses are highly variable, with some patients showing large treatment effects and others little to none with no way for clinicians to distinguish between those who will and will not respond. A biomarker predictive of cardio-metabolic response would be particularly helpful for minimally symptomatic OSA patients who will not accept CPAP treatment unless some benefits in terms of risk reduction can be monitored. It has been reported in cohort studies that CPAP treatment is associated with an increase in IGF-1 levels (42, 44, 54) and is related to adherence to CPAP therapy (55); however, in a parallel randomized sham-placebo controlled 1-month trial, both therapeutic and sub-therapeutic nasal CPAP improved IGF-1 without differences between the groups (56). Therefore, whether CPAP is able to restore the somatotropic axis and IGF-1 levels need further controlled studies and whether an increase in IGF-1 under CPAP is related to long-term cardiovascular protection remains to be established.

### Study Strengths and Limitations

Our study was by far the largest in the field including in 10 sleep centers in private and academic practices more than 800 patients referred for OSA suspicion. We acknowledge the heterogeneity in methods for IGF-1 assays but this was mandatory for assuring the feasibility of the study and the assessment of a unique large sample of patients. The major study strength was the capability to assess an unbiased population but this is balanced by a restricted dataset regarding the respective distribution of the different sleep stages namely slow wave sleep which is classically related to IGF-1 levels. IGF-1 levels vary with age but we have adjusted the multivariate analysis with age. Data are also limited regarding indices of insulin resistance as circulating insulin or HBA1C were not systematically assessed. Further studies are needed to assess IGF-1 changes after OSA treatment. The reliability of IGF-1 as a potential marker of cardiovascular risk in OSA patients remains to be validated in longitudinal studies.

## Conclusion

In a large multicenter OSA population, Low IGF-1 levels were associated with severity of apnea+ hypopnea index, measures of adiposity and lipids abnormalities. Both adiposity and sleep apnea could synergistically contribute toward higher cardio-metabolic risk. The risk of low IGF-1 levels increased with OSA severity. We suggest that IGF-/1 might be helpful in OSA personalized medicine for grading severity cardio-metabolic risk in OSA and guiding specific interventions.

## Data Availability Statement

The datasets generated for this study are available on request to the corresponding author.

## Ethics Statement

The studies involving human participants were reviewed and approved by Grenoble Alpes University Hospital ethics committee (IRB: 13-CHUG-37). The database was approved by “Le Comité consultatif sur le traitement de l'information en matière de recherche en santé” (C.C.T.I.R.S n° 09.521) with authorization from the “Commission Nationale Informatique et Liberté” (C.N.I.L), the French information technology and personal data protection authority. The patients/participants provided their written informed consent to participate in this study.

## Author Contributions

L-MG and A-LB analyzed the data and wrote the manuscript. J-LP designed the study, analyzed data, and critically reviewed the present work. OC, PC, MS, BS, JG-R, and RT participated in the conception of the study design and critically reviewed the manuscript.

## Conflict of Interest

OC has received invitations from IPSEN to scientific meetings. J-LP has received a lecture fee from IPSEN. The remaining authors declare that the research was conducted in the absence of any commercial or financial relationships that could be construed as a potential conflict of interest. The reviewer MB declared a past co-authorship with the authors L-MG, RT, and J-LP to the handling editor.
